# Frequency of metabolic abnormalities in urinary stones patients

**DOI:** 10.12669/pjms.296.4007

**Published:** 2013

**Authors:** Iftikhar Ahmad, Mudassar Saeed Pansota, Muhammad Tariq, Shafqat Ali Tabassum

**Affiliations:** 1 Dr. Iftikhar Ahmad, FCPS, Assistant Professor, Department of Urology and Renal Transplantation, Bahawal Victoria Hospital/Quaid-e-Azam Medical College, Bahawalpur.; 2 Dr. Mudassar Saeed Pansota, Post Graduate Resident, Department of Urology and Renal Transplantation, Bahawal Victoria Hospital/Quaid-e-Azam Medical College, Bahawalpur.; 3 Dr. Muhammad Tariq**, **FCPS, Medical Officer, Department of Urology and Renal Transplantation, Bahawal Victoria Hospital/Quaid-e-Azam Medical College, Bahawalpur.; 4 Prof. Dr. Shafqat Ali Tabassum**, **FCPS, Head, Department of Urology and Renal Transplantation, Bahawal Victoria Hospital/Quaid-e-Azam Medical College, Bahawalpur.

**Keywords:** Urolithiasis, Metabolic evaluation, Hyperoxaluria

## Abstract

***Objective:*** To determine the frequency of metabolic abnormalities in the serum and urine of patients with urinary stones disease.

***Methods:*** Two hundred patients with either multiple or recurrent urolithiasis diagnosed on ultrasonography and intravenous urography were included in this study. 24 hour urine sample were collected from each patient and sent for PH, specific gravity, Creatinine, uric acid, calcium, phosphate, oxalate, citrate and magnesium. In addition, blood sample of each patient was also sent for serum levels of urea, creatinine, uric acid, phosphate and calcium.

***Results:*** Mean age of patients was 38 ± 7.75 years with male to female ratio of 2:1. The main presenting complaint was lumber pain and 82.5% patients were found to have calcium oxalate stones on chemical analysis. Metabolic abnormalities were found in 90.5% patients, whereas there were no metabolic abnormalities in 19 (9.5%) patients. Forty patients (21.5%) only had one metabolic abnormality and 157 (78.5%) patients had multiple metabolic abnormalities. Hyperoxaluria was the most commonly observed metabolic abnormality and was found in 64.5% patients. Other significant metabolic abnormalities were hypercalciuria, Hypercalcemia, hypocitraturia and hyperuricemia.

***Conclusion:*** This study concludes that frequency of metabolic abnormalities is very high in patients with urolithiasis and hyperoxaluria, hypercalciuria and hypocitraturia are the most important metabolic abnormalities observed in these patients.

## INTRODUCTION

Urolithiasis is a common disease throughout the world that affects the general population. It is estimated that approximately 2% of the population experience renal stone disease at sometimes in their life with peak incidence in 2^nd^ and 3^rd^ decades of life.^1^ There are several types of urinary stones, and they are classified according to chemical composition. Calcium oxalate is the major component of the vast majority of stones.^2^ Several factors, such as age, gender, climate, metabolic abnormalities and heredity, are related to the development of urinary stones.^3^ Metabolic abnormalities are the most important factors because they can be modified to prevent the risk of urinary stones.^4^

The evaluation of patient with urolithiasis consists of radiographic imaging along with blood and urine testing. Although there is a general agreement that a complete metabolic evaluation is indicated in all patients with stone formation, some studies have shown medical evaluation is not cost effective for patients who have only formed one stone.^5^

Metabolic abnormalities such as hypercalciuria, hyperuricosuria, hypokalemia, hyperuricemia, hypophosphatemia and low urine volume that cause stone disease varies in different population and environmental and genetic factors might result in these differences.^6^ In patients with urinary stones, metabolic evaluation and intervention should be considered to prevent the recurrence of stones. Moreover, calcium stone formers, those who have recurrent stones, those who have multiple stones or bilateral stones at their first presentation and children with a stone history should always have an evaluation as a correctable abnormality is often found in these patients.^7^^,^^8^

The purpose of this study was to determine frequency of metabolic abnormalities in urinary stones patients in local population, so that a management policy could be designed for urinary stones patients to prevent stone recurrence.

## METHODS

This cross-sectional study was conducted at the Department of Urology & Renal Transplantation, Bahawal Victoria Hospital/Quaid-e-Azam Medical College, Bahawalpur from June 2011 to December 2012. After taking permission from ethical review committee, total number of 200 patients with either multiple or recurrent urolithiasis diagnosed on ultrasonography and intravenous urography were included in this study. Patients with single stone, urinary disorders i.e. proteinuria, recurrent urinary tract infection, congenital urinary tract obstruction and bladder outflow obstruction and with any chronic disease i.e. chronic renal failure, chronic liver disease and with history of any chronic drug usage were excluded. Patients with any known metabolic abnormality and hypo- or hyperthyroidism were also excluded from the study.

After taking informed consent and relevant history, 24 hour urine sample was collected from each patient and sent for PH, specific gravity, Creatinine, uric acid, calcium, phosphate, oxalate, citrate and magnesium. Twenty-four hour urine samples were collected in plastic boxes, which do not react chemically by standard methods, and were stored at 2-8ºC. In addition, blood sample of each patient was also sent for serum levels of urea, creatinine, uric acid, phosphate and calcium. The serum levels of metabolic parameters were measured by standard chemical procedures. All patients then had definitive procedure after completion of all workup and stones were sent to pathology laboratory for chemical analysis to know about the stone composition.

Statistical analysis was performed using SPSS version 16.0. Mean and standard deviation was calculated for quantitative variables and frequency and percentage was calculated for qualitative variables. Chi square was applied and p value ≤ 0.05 was considered as significant.

## RESULTS

Age range in this study was from 10 to 50 years with mean age of 38 ± 7.75 years. Majority of the patients 79 (39.5%) were between 31 to 40 years of age as shown in Table-I. Out of the 200 patients, 135 (67.5%) were male and 65 (32.5%) were females with ratio of 2:1.

The main presenting complaint was lumber pain on the affected side i.e. in 79.0% patients, followed by hematuria and burning micturation. Majority of the patients i.e. 94.0%, were diagnosed as having renal stone or ureteric stone. Only 38.0% patients presented with recurrent stones while remaining 62.0% had stone for the first time. Chemical analysis of stones after definitive procedure had shown calcium oxalate stone in 82.5% patients. Descriptive statistics for different variables have been shown in Table-II.

Metabolic abnormalities were found in 181 (90.5%) patients, whereas there were no metabolic abnormalities in 19 (9.5%) patients. Forty patients (21.5%) only had one metabolic abnormality, and 157 (78.5%) patients had multiple metabolic abnormalities. Hyperoxaluria was the most commonly observed metabolic abnormality and was found in 129 (64.5%) patients. Other significant metabolic abnormalities were hypercalciuria, Hypercalcemia, hypocitraturia and hyperuricemia as shown in Table-III.

**Table-I:** Percentage of patients according to age group (n=200).

*Age (years)*	*Male*		*Female*		*Total*	
	*No. of patients*	*%age*	*No. of patients*	*%age*	*No. of patients*	*%age*
10-20	15	7.5	08	4.0	23	11.5
21-30	22	11.0	13	6.5	35	17.5
31-40	52	26.0	27	13.5	79	39.5
41-50	46	23.0	17	8.5	63	31.5
Total	135	67.5	65	32.5	200	100.0

**Table-II:** Descriptive statistics for different variables (n=200).

*Features*	*No. of patients*	*%age*
*Presenting Complaint:*		
Lumber pain Hematuria Burning micturation	158 26 16	79.0 13.0 8.0
*Diagnosis:*		
Renal stone Ureteric stone Renal + Ureteric stone Urinary bladder stone	126 42 20 12	63.0 21.0 10.06.0
*Recurrent stone:*		
Yes No	76 124	38.0 62.0
*Family history of Urolithiasis:*		
Yes No	128 72	64.0 36.0
*Stone composition on Stone analysis:*		
Calcium oxalate Calcium phosphate Uric acid Struvite Cystine	165 05 23 03 04	82.5 2.5 11.5 1.5 2.0

**Table-III:** Frequency of Metabolic abnormalities

*Metabolic abnormality*	*Frequency*	*%age*
Hyperoxaluria (oxalate > 45 mg/d)	129	64.5
Hypercalciuria (> 250 mg/d for women and > 300 mg/d for men)	87	43.5
Hypocitraturia (citrate levels < 320 mg/d)	81	40.5
Hypernatriuria (sodium level > 220 mmol/ day)	59	29.5
Hyperuricosuria ( > 600 mg/d in women and > 750 mg/d in men)	43	21.5
Hypomagnesuria (magnesium level < 3 mg/day)	27	13.5
Hyperphosphaturia (phosphate level > 1.3 g/day)	23	11.5
Hypercalcemia (calcium above the normal range i.e. 8.4-10.2 mg/dl):	93	46.5
Hyperuricemia: (normal range 2.5-8 mg/dL for males and 1.5-6.0 mg/dL for females).	59	29.5

## DISCUSSION

Advances in minimally invasive techniques have dramatically changed surgical management of stone disease in the last trwo decades. Urolithiasis can develop as a result of metabolic disorders, anatomical malformations of the urinary tract, infection, and environmental and nutritional factors.^2^ Therefore, not only genetic and environmental factors, but also metabolic ones are implicated in the pathogenesis of stone formation.^9^ Metabolic evaluation of recurrent stone formers not only identify the abnormality but also helps in determining the drug choices and dosages.^6^

The mean age of patients in this study was 38 ± 7.75 years which was a little lower than Kirac M et al^4^ and Majalan NN et al^10^ studies who had found mean age of 42 and 43 years respectively. Urolithiasis is a disease that is known to predominantly affect males. In recent years, however, the incidence of urolithiasis in women has been increasing. A study by Scales et al^11^ found that the male:female ratio of urolithiasis cases diminished from 1.7 to 1.3 during the last 20 years in the USA. In this study, we found a male predominance (i.e., the male:female with ratio of 2:1) which is very much comparable to studies of Parvin M et al^6^ and Kirac M et al^4^ who had also found predominance of male patients with urolithiasis.

In our study, the main presenting complaint was lumber pain i.e. in 79.0% patients. Elfadil GA et al^7^ had also found flank pain as the chief presenting complaint in his study i.e. in 67% patients. The results of our study have shown a strong genetic predisposition to urinary stone disease as 64.0% patients had family history of urolithiasis. This genetic factor is also supported by studies of Kirac M et al^4^ and Majalan NN et al^10^ who had found a positive family history in 67.0% and 53.1% patients respectively. On the other hand, Elfadil GA et al^7^ had found this in only 20% of their patients. We had also found 38.0% patients with recurrent urinary stones and the major stone component was calcium oxalate in our study which was also found by Elfadil GA et al.^7^ But in a study by Androulakakis et al^12^, the main components of urinary stones in Europe, in decreasing order, are struvite, calcium phosphate and calcium oxalate.

In our study, metabolic abnormalities were found in 90.5% patients, whereas there was no metabolic abnormality in only 9.5% patients which is very much comparable to many previous studies.^4^^,^^6^^,^^8^^,^^10^ In a study by Amaro et al^8^, 62.2% of patients had multiple metabolic abnormalities; however, the patients did not have recurrent calcium oxalate stones. Therefore, it can be presumed that multiple metabolic abnormalities are more common in patients with recurrent calcium oxalate stones. Kirac M et al^10^ in his study had found multiple metabolic abnormalities in 71.3% patients while in our study, 78.5% patients had multiple metabolic abnormalities and only 21.5% had one metabolic abnormality.

Hyperoxaluria was the most commonly observed metabolic abnormality in this study and was found in 64.5% patients. Interestingly, the rate of hyperoxaluria is not consistent between studies that have evaluated metabolic abnormalities i.e. Kirac M et al^4^ reported a 64.4% prevalence of hyperoxaluria, whereas Amaro et al^8^ found a 21% prevalence. Parvin M et al^6^ have also reported higher mean urinary excretion of oxalate in stone formers compared with the individuals who are not stone formers. Although hyperoxaluria was the most common risk factors in many previous studies but it was the second most common risk factor in the study by Hess and associates,^13^ and also not very common in the Thai stone formers.^14^

Other significant metabolic abnormalities as observed in our study were hypercalciuria and hypocitraturia. Hypercalciuria was found in 43.5% patients in this study while Majalan NN et al^10^ and Kirac M et al^4^ had found its prevalence as 27% and 32.8% respectively. Citrate is a natural inhibitor of stone formation, and its absence in urine causes an increase in the risk of stone formation. Numerous studies have shown that the prevalence of hypocitraturia is between 19% and 63%.^4^^,^^6^^,^^10^^,^^14^^,^^15^ In the present study, the hypocitraturia was found in 40.5% patients.

Hypernatriuria was found in 29.5% of the patients in this study. Therefore, urinary sodium should also be evaluated along with calcium, oxalate and citrate in patients with recurrent calcium oxalate stones. In recurrent stone patients, urinary sodium has hypocitraturic and calciuric effect.^4^ In a study by Brockis et al^16^, hyperuricosuria and hyperuricemia were the most common metabolic changes, and these conditions were more commonly observed in males. In a study by Kirac M et al^4^ the prevalence of hyperuricosuria was 14.7%. Similarly, we have also found hyperuricosuria in 21.5% patients and hyperuricemia in 29.5% patients which showed that hyperuricosuria and hyperuricemia are also common metabolic abnormalities in urolithiasis.

## CONCLUSION

This study concludes that frequency of metabolic abnormalities is very high in patients with urolithiasis and hyperoxaluria, hypercalciuria and hypocitraturia are the most important metabolic abnormalities observed in these patients. Therefore, we recommend that metabolic evaluation of every urinary stone former should be done especially in calcium stone formers, those who have recurrent stones, multiple stones or bilateral stones at their first presentation and in children. It will not only help us in recognizing urolithiasis risk factors, but also for selection of proper medical and dietary therapies to prevent recurrent stone formation.

## Authors’s Contribution:

IA and MSP: Conception and Design, acquisition of data, analysis and interpretation of data, drafting and critical revision, final approval of the version to be published. MT: Acquisition of data, drafting and final approval of the manuscript. SAT: Conception, acquisition of data, critical revision of the manuscript.
